# Implementing a quality management system using good clinical laboratory practice guidelines at KEMRI-CMR to support medical research

**DOI:** 10.12688/wellcomeopenres.14860.2

**Published:** 2019-06-25

**Authors:** Horace Gumba, Joseph Waichungo, Brett Lowe, Alfred Mwanzu, Robert Musyimi, Johnstone Thitiri, Caroline Tigoi, Martin Kamui, James A. Berkley, Ronald Ngetich, Susan Kavai, Samuel Kariuki

**Affiliations:** 1 KEMRI-Wellcome Trust Research Programe, Kilifi, Coast, 80108, Kenya; 2 Centre for Tropical Medicine and Global Health, University of Oxford, London, UK; 3 The Childhood Acute Illness & Nutrition (CHAIN) Network, Nairobi, Kenya; 4 KEMRI-Centre for Microbiology and Research, Nairobi, Kenya

**Keywords:** Good Clinical Laboratory Practice, Quality Assurance, Quality system, medical research, quality management system.

## Abstract

**Background: **Good Clinical Laboratory Practice (GCLP) is a standard that helps ensure the quality and reliability of research data through principles of Good Laboratory Practice (GLP) and Good Clinical Practice (GCP). The implementation of GCLP includes careful documentation of procedures, competencies and safety measures. Implementation of GCLP is influenced by existing resources and quality systems, thus laboratories in low- and middle-income countries may face additional challenges.

**Methods: **This paper describes implementation of GCLP at the Kenya Medical Research Institute-Center for Microbiology Research (KEMRI-CMR) as part of a quality system to support medical research. This study employed assessment, twinning (institutional mentorship) model, conducting relevant training workshops and Kaizen 5S approaches to implement an effective quality management system using GCLP standard. This was achieved through a collaboration between the KEMRI/Wellcome Trust Research Programme (KWTRP) and KEMRI-CMR. The aim was compliance and continuous monitoring to meet international GCLP standards in a way that could be replicated in other research organizations.

**Results: **Following a baseline assessment in March 2017, training, mentorship and a cycle of quality audit and corrective action using a Kaizen 5S approach (sorting, setting in order, shining, standardizing and sustaining) was established. Laboratory personnel were trained in writing standard operating procedures and analytical plans, microbiological techniques, and good documentation practice. Mid-term and exit assessments demonstrated significant declines in non-conformances across all GCLP elements. KEMRI-CMR achieved GCLP accreditation in May 2018 by Qualogy Ltd (UK).

**Conclusions: **Involving all the laboratory personnel in implementation of quality management system processes is critical to success. An institutional mentorship (twinning) approach shows potential for future collaborations between accredited and non-accredited organizations to accelerate the implementation of high-quality management systems and continuous improvement.

## Introduction

Medical laboratories play an important role in disease diagnosis, treatment guidance, drug resistance monitoring and surveillance of diseases of public health interest (Gershy & Rotz, 2010; Martin *et al*., 2005; Wians, 2009). According to Nkengasong (2010), 20% of clinical trials in Africa have been suspended due to serious Good Clinical Practice (GCP) breaches, which mainly impact on participants’ safety and reliability of the data generated. This can be addressed by implementing integrated, tiered and harmonized operations, and a well-functioning laboratory quality system ( Nkengasong, 2010). Moreover, the emergence of the recent Ebola virus disease epidemic in West Africa in 2015 emphasized the need to rapidly develop better laboratory systems that will foster increased accuracy and reliability of the data generated ( Gostin *et al.*, 2015; Heymann *et al*., 2015), which have often been the traditional meaning of quality in medical laboratories ( Harteloh, 2004). In medical research, it is imperative to note that generation of reproducible and re-constructible results can be achieved when the clinical laboratory operates under a robust and mature quality management system (QMS) that complies with the GCLP standards, thus providing an excellent path for the success of conducting medical research.

## What is the GCLP standard?

GCLP is a standard that supports both the research and clinical aspects of Good Laboratory Practice ( Ezzelle *et al*., 2008). It was developed to support and strengthen research laboratories performing human clinical trials and provides a platform for monitoring the global conduct of clinical laboratory work performed under harmonized operations (Marcella *et al*., 2009). This standard was developed by merging the principles of Good Clinical Practice and Good Laboratory Practice in conjunction with the regulatory authorities and accrediting bodies, and was the same approach adopted by the British Association of Research Quality Assurance (BARQA) to develop the Good Clinical Laboratory Practice standard ( Stiles *et al.,* 2003). The GCLP standard focusses on the building blocks of a quality system, which includes assessments, assay validation and verification, training of personnel involved in the research, organization and personnel, specimen management, laboratory equipment, reagents, records and reports, laboratory safety, quality control and proficiency testing programmes, laboratory information systems, and the overall quality management plan of the laboratory (Marcella *et al*., 2009). The expectation of implementing the GCLP quality system is that data of high quality will be generated when the laboratory complies to the GCLP guidelines. In addition, it provides guidance on the development of a quality system that ensures integrity, validity and reliability of clinical trials data.

## The Kenya Medical Research Institute – Centre for Microbiology and Research (KEMRI-CMR)

The Kenya Medical Research Institute (KEMRI) is a Kenyan government parastatal that regulates and conducts research in human health with the aim of improving wellbeing, and the formulation and implementation of policy formulation, while collaborating with other global research organizations. It has its centers widely spread around the country that perform research focusing on different fields ( KEMRI, 2018). Even though KEMRI is the leading medical research organization in the country, some of its centers do not have up-to-date quality systems in place to support medical research. There is an urgent need to establish an effective quality management system using GCLP guidelines to support clinical trials and other studies.

KEMRI-CMR, based in Nairobi, is one of the oldest KEMRI research centers. Research has focused predominantly on traditional and molecular characterization of enteric pathogens in communities and in hospital attendees, in addition to their transmission, virulence and antimicrobial profiles. To promote and support its research activities, KEMRI-CMR engaged its sister organization KEMRI-Wellcome Trust Research Programme (KWTRP), to assist in the development of a quality system using GCLP guidelines. The KWTRP, based in Kilifi, has been actively undertaking microbiological research since 1992, predominantly on invasive bacterial infections in children, including surveillance and antimicrobial treatment trials. KWTRP has been GCLP-accredited since 2007. Here, we describe how the quality management system was implemented at KEMRI-CMR using GCLP guidelines to support medical microbiological research with the goal of gaining recognition of the quality of their management system by attaining GCLP accreditation. The GCLP standards developed by BARQA was selected for this project because the mentor laboratory (KWTRP) had been accredited using this GCLP standard and it would be easier to replicate the same in the mentee laboratory (KEMRI-CMR).

## Methods

### Methodology used

This study employed assessment, twinning (institutional mentorship) model ( Makokha *et al*., 2014), and conducting training workshops to build a competent laboratory workforce and utilizing Kaizen 5S approaches to implement an effective quality management system using GCLP standards ( Stiles *et al*., 2003).

### Baseline, mid-term and exit assessments

The QMS implementation progress was evaluated by performing assessments using a GCLP accreditation audit checklist, developed by Qualogy Ltd UK ( Qualogy, 2018) ( Table 1). This checklist consists of 12 sections of 15 questions, which covered the entire quality system elements defined by GCLP guidelines obtained from Qualogy, Ltd, and had a total score of 270 points. The audit checklist questions were asked by the mentors from KWTRP to the auditees (laboratory staff from KEMRI-CMR).

**Table 1.** Quality management system elements and their scores on the good clinical and laboratory practice (GCLP) accreditation checklist. Source: Stiles *et al*., 2003.

GCLP Elements	Total points
**Section 1:** Documents, records and reports	30
**Section 2**: Trial samples and management	14
**Section 3**: Organization and personnel	22
**Section 4**: Reporting of results	10
**Section 5**: Equipment, materials and reagents	33
**Section 6**: Internal audits and corrective action	15
**Section 7**: Retention and archiving of records	32
**Section 8**: Quality control and external quality assessment	21
**Section 9**: Planning of the work & sub-contracting	17
**Section 10**: Conduct of work	24
**Section 11**: Confidentiality, blinding and patient safety	12
**Section 12**: Facilities and safety	40
**TOTAL**	**270**

In total, three assessments were performed throughout the process to establish the laboratory’s performance and progress towards GCLP accreditation, as well as determining any remaining gaps. In March 2017, a week after the initial engagement, a baseline assessment was conducted at KEMRI-CMR, using the GCLP accreditation checklist ( Qualogy, 2018). This assessment was performed by the laboratory quality officer (mentor, from KWTRP and its results provided the basis for developing KEMRI-CMR-specific actions. A mid-term assessment was conducted 3 months (June 2017) after the baseline assessment, following a GCLP training workshop and corresponding GCLP assignment elements assessed using the GCLP accreditation checklist developed by the mentor laboratory (KWTRP). The exit assessment was performed three months (October 2017) after the mid-term assessment by an independent auditor from KWTRP who was not involved in the training, and was the final assessment in readiness for the GCLP accreditation audit by Qualogy UK Ltd.

### Twinning (institutional mentorship) model

The twinning (institutional mentorship) model was also employed to implement QMS ( Makokha *et al.,* 2014). This was conducted during the period of May-June 2018. Using this model, a total of 24 laboratory staff from the mentee laboratory (KEMRI-CMR) were paired to the mentor laboratory (KWTRP) to learn and subsequently implement GCLP processes in their laboratory upon their return. A total of 12 laboratory staff from KEMRI-CMR were twinned with staff from KWTRP in the month of May 2017 and another 12 laboratory staff twinned in June 2017. To facilitate the twinning relationship, the laboratory quality officer (mentor) spent 1 week at the mentee laboratory to provide mentorship and coaching for the GCLP process.

### Conducting KEMRI-CMR laboratory training

The QMS mandatory training and other relevant training workshops were identified with the aim of strengthening knowledge, skills and abilities, and changing attitudes. The training was mainly delivered through workshops, coaching, and visits to KWTRP for a period of 2 weeks. Training was delivered by the lead mentor and two co-mentors. Subjects of the training sessions, alongside the trainer and the dates of training, are listed in Table 2. Once these sessions were complete, staff were assigned a specific area to implement when they go back to their laboratory. Kaizen 5S ( Kobayashi, 2005) was implemented to establish the foundation for continuity of quality management system at KEMRI-CMR.

**Table 2.** Training sessions given to staff.

Training	No. of staff trained	Trainers	Training dates
Microbiological techniques	25	Lead mentor & co-mentors	25–28 April 2017
KWTRP Exchange visits	24	Attached to section heads of KWTRP	1 ^st^ group: 2–12 May 2017 2 ^nd^ group: 19–30 June 2017
Good documentation Practice	25	Lead mentor	16 June 2017
SOP writing training	25	Lead mentor	7–9 June 2017
Improvement projects and Quality Indicator training	10	Lead mentor	6–7 July 2017
Confidentiality, blinding and patient safety monitoring	12	Lead mentor (myself) & co-mentors (Joseph & Robert)	20–21 July 2017
Method and equipment validation	10	Lead mentor	24–25 August 2017
Analytical plan writing training	26	Lead mentor	6–8 September 2017
Internal audits	6	Lead mentor	8–11 August 2017
Basic GCLP training	30	Lead mentor	11–14 April 2017

KWTRP, KEMRI-Wellcome Trust Research Programme; SOP, standard operating practice; GCLP, good clinical and laboratory practice.

### Data analysis

Data from the three assessments, training conducted were analyzed using Microsoft Excel and presented in tables and figures to extract their useful meaning.

## Results

### KEMRI-CMR performance

**Figure 1.**
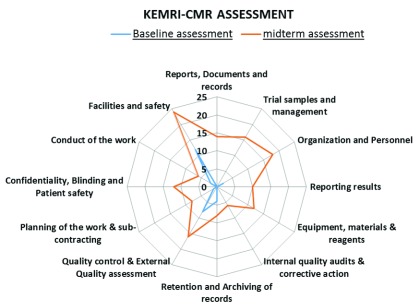
Comparison between baseline and mid-term assessments. KEMRI-CMR, Kenya Medical Research Institute – Centre for Microbiology Research.

**Figure 2.**
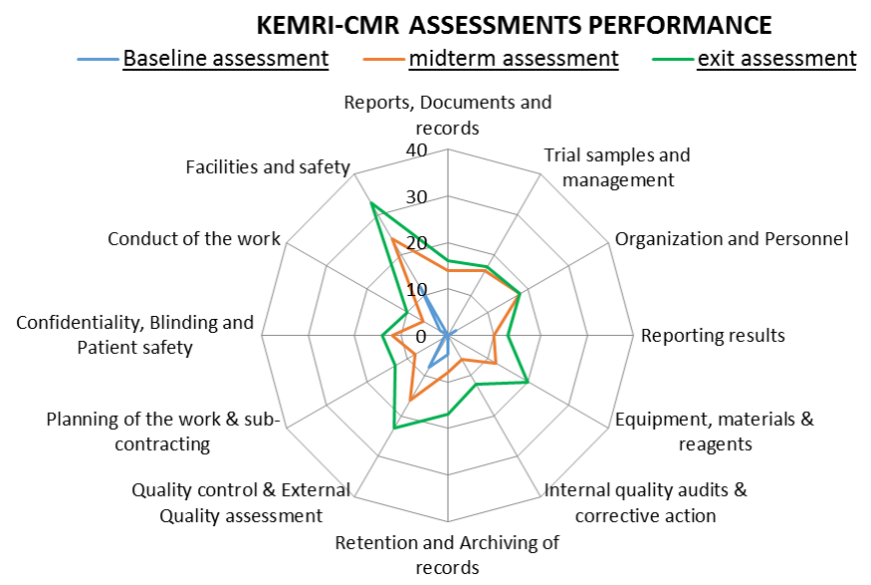
Kenya Medical Research Institute – Centre for Microbiology Research (KEMRI-CMR) performance comparison of the 12 good clinical and laboratory practice elements between the three assessments.

The KEMRI-CMR laboratory assessment performance is summarized in Figure 1 and Figure 2. All 12 elements in the GCLP accreditation checklist were improved at successive assessments ( Figure 1 and Figure 2). The most improved element was the facilities and safety element, followed by quality control, external quality assessment and equipment, reagents and materials elements. The laboratory performed less well in the reporting of results, conduct of the work, internal quality audits, corrective action, planning of the work and sub-contracting GCLP elements.

**Figure 3.**
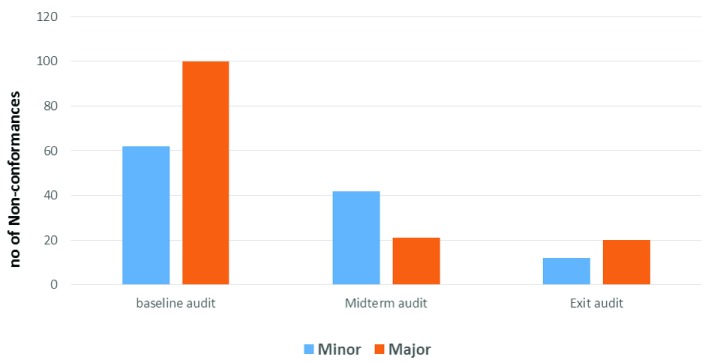
Non-conformance analysis at the baseline, midterm and exit assessments.

A total of 162 non-conformances arose from the baseline assessment (100 major findings and 62 minor findings); 62 non-conformances arose in the mid-term assessment (42 major findings & 20 minor findings); and 32 non-conformances arose in the final exit assessment (20 major findings & 12 minor findings). The decrease in major and minor non-conformities indicated progress in resolving queries and implementing corrective action ( Figure 3).

### KEMRI-CMR laboratory training

**Figure 4.**
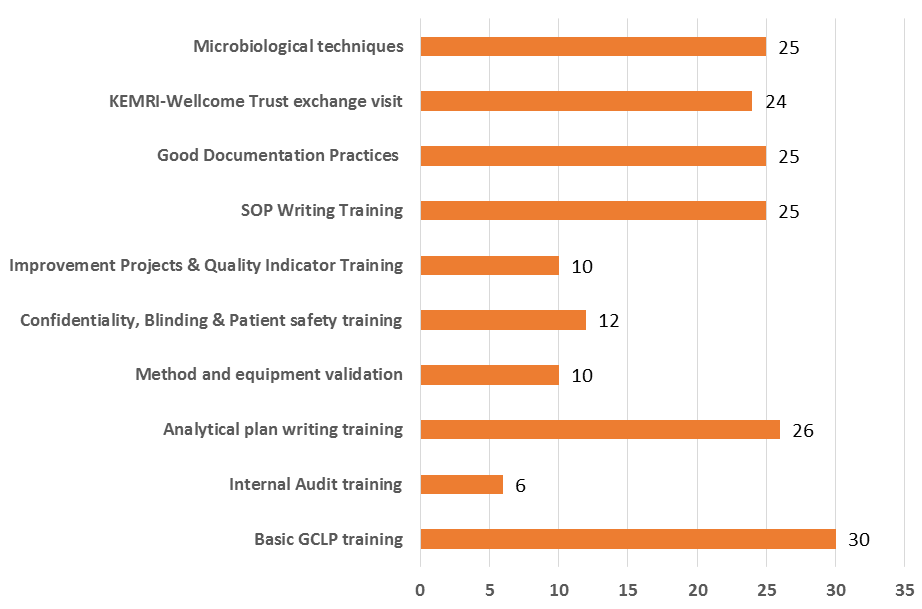
Number of staff trained in the indicated area during the mentorship period. SOP, standard operating practice; GCLP, good clinical and laboratory practice.

To build a competent and skilled laboratory workforce in the KEMRI-CMR laboratory, a total of 10 training sessions and workshops were conducted between April 2017 and September 2017. These trainings aimed to strengthen the quality of services and systems in KEMRI-CMR. GCLP training was provided ( Table 2) to twenty-five laboratory personnel. In total, 9 of the 10 conducted trainings were done onsite to allow more staff to attend and to reduce costs. All 25 (100%) laboratory personnel were trained in writing SOPs and analytical plans, microbiological techniques, and good documentation practice ( Figure 4).

To decongest the laboratory and enhance efficient workflow for productivity management, principles of Kaizen 5S were adopted: equipment was rearranged for optimal workflow while removing obsolete and un-wanted materials from the laboratory. Equipment that was close to sinks was removed and placed separately as per the specimen workflow. The removal of obsolete equipment and old records that consumed considerable space enhanced the efficiency of the workflow. Documentation was done before and after the Kaizen 5S for comparison. KEMRI-CMR achieved a GCLP accreditation in May 2018 by Qualogy Ltd (UK).

## Discussion

The results from the baseline to exit GCLP assessments showed the greatest improvements in facilities and safety element (21 points), followed by quality control and external quality assessment (15 points) and equipment, reagents and materials elements (14 points). The areas that were more challenging to improve were internal quality audits and corrective action (an improvement of 6 points), conduct of work (an improvement of 4 points) reporting of results and planning of the work (improvement of 3 points). The slower progress of these GCLP elements were attributed to unfamiliarity with the internal audit system by the laboratory staff, characterized by inadequate follow-up of the internal audit findings and insufficient documentation of corrective actions (management reviews as described by ISO 15189 standards) described by Maina *et al*. (2014) as Factor X. Despite the less strong performance in these three GCLP elements, the KEMRI-CMR laboratory QMS performance improved steadily from 10.7% at baseline assessment to successfully achieving 76.3% at the exit assessment.

The improvement of KEMRI-CMR’s laboratory QMS performance was in a large part due to staff’s positive attitude and commitment to work, and continued senior management support. Despite an initial opposition to change, there was a great enthusiasm to continue improving laboratory performance as observed in the final assessment results. Clearly identifying gaps and involving all staff in frank discussions about their solutions was key to achieving this. The foundation of best practice, and a ‘quality culture’ were established through the exchange visits, conducting trainings, mentors’ assistance coupled with managerial commitment. This reflects reports from other institutions implementing quality management process ( Andiric & Massambu, 2014). 

The implementation of Kaizen 5S greatly improved the laboratory’s workflow and space. The results indicate that there is a strong foundation for continuity of the quality management system at KEMRI-CMR ( Khamis *et al*., 2009). The entire laboratory was physically re-organized by placing the equipment strategically to improve efficiency and enhance safety. The entire Kaizen 5S methodology for this study provided the best platform to accelerate the process of quality improvement process at KEMRI-CMR.

Engaging the management team of KEMRI-CMR through the leadership of the Centre Director was crucial in securing financial support for renovating the laboratory and providing adequate human resources for the quality implementation process. His open-door policy style of management and having frequent discussions with the laboratory staff and the mentors made him clearly understand the significance of implementing a quality management system. Moreover, the formation of the fortnightly laboratory meetings to provide reports, feedback and recommendations accelerated the implementation of the quality management system using GCLP guidelines.

Conducting training on-site has also been shown to be an improvement factor during QMS implementation ( Nkwawir *et al*., 2014). The trainings conducted at KEMRI-CMR laboratory coupled with twinning of the KEMRI-CMR laboratory staff to the KWTRP through exchange visits also accelerated the implementation of a QMS. Conducting mandatory (Basic GCLP, SOP writing, confidentiality, blinding and patient safety monitoring and analytical plan writing) and supplementary QMS training to cover best laboratory practices within the KEMRI-CMR laboratory led to more staff being trained ( Figure 4).

Using the twinning model or the institutional mentorship approach ( Makokha *et al*., 2014) helped the mentor to more fully understand the operational functionality of the mentee laboratory by participating in the laboratory activities, providing hands-on trainings and guidance regarding what aspects of the quality management system to be implemented.

In addition, the continued presence of the mentors at the KEMRI-CMR laboratory during the entire QMS implementation period helped to design specific activities tailored in their approach to assisting laboratory improvements, developing a working culture that emphasizes quality and a sustainable QMS as previously implemented by other organizations during their QMS journey ( Nkwawir *et al*., 2014). Only one training (GCLP training) was attended by staff drawn from other departments. This was to enhance their understanding of the GCLP concept so that they could support the laboratory’s journey of implementing the quality management system. The experience at KEMRI-CMR during the quality implementation process clearly reveals what other laboratories that fully commit their concerted effort can achieve in implementing a quality improvement process.

## Conclusions

Implementing an efficient and effective quality management system requires a system-wise approach and strong teamwork to ensure that set goals and objectives are realized. Compliance with GCLP standards, coupled with periodic audits/assessments, will help ensure that clinical research and trials performed at KEMRI-CMR meets international standards. Involving all laboratory personnel in the implementation of a QMS process is critical to its success. The use of an institutional mentorship (twinning) approach also shows the potential for future collaborations between accredited and non-accredited organizations and can be used to accelerate the implementation of a good QMS and continuous improvement.

## Data availability

Data generated in the present study are available on figshare, DOI: https://doi.org/10.6084/m9.figshare.7200707 ( Gumba, 2018).
