# Supplementary material for: Implementing a quality management system using good clinical laboratory practice guidelines at KEMRI-CMR to support medical research
Source: Wellcome Open Res. 2019 Jun 25;3:137. Originally published 2018 Oct 31. [Version 2] doi: 10.12688/wellcomeopenres.14860.2 (PMC6305232; doi:10.12688/wellcomeopenres.14860.2)
Supplement: Peer review history

# Peer review: Reviewer response for version 1

**DOI:** 10.21956/wellcomeopenres.16195.r34150

**Published:** 2018-12-19

**Authors:** Paramesh Chetty

**Affiliations:** 1 Human Immunology Laboratory (HIL), International AIDS Vaccine Initiative (IAVI), London, UK

1. The author mentions that GCLP is a standard when it is a guideline. Need to give some background as to why BARQA GCLP guideline was adopted.
2. Give more mention on resources as to implement the GCLP guidelines for GCLP compliance as mentioned it ensures all documentation are in place that must be followed to ensure GCLP compliance. This requires basic resources, unless the facility is relatively under-staffed.
3. Elaborate on the baseline assessment and disaggregate the non-conformances to the categories or topics of the training offered. This will give a clearer view of what non-conformances were resolved at exit visit and what was not.
4. There must be more background on why the Kaizen 5S approach was used and if this approach was also used at the KEMRI-Kilifi facility and proved to be successful.
5. Table 1: Give the details of the sections and the total points, but not more information on how the point scoring worked and was it related to findings. More information required on the types of findings (Differentiate between major and minor and advise what is acceptable to be GCLP compliant). Do a comparison between baseline, mid-term and exit audit against the findings to conclude if the facility is GCLP compliant as there are still higher major findings than minor at exit audit.
6. Need to be clearer in conclusion as to the outcome and impact and not only the output (e.g. Numbers only of those staff trained and non-conformances identified). Even though the non-compliances were reduced, did the non-conformances at the exit visit still impact the GCLP compliance of the site?

I confirm that I have read this submission and believe that I have an appropriate level of expertise to confirm that it is of an acceptable scientific standard.

# Peer review: Reviewer response for version 1

**DOI:** 10.21956/wellcomeopenres.16195.r34152

**Published:** 2018-12-18

**Authors:** Robert Njuguna

**Affiliations:** 1 Kenya Accreditation Service (KENAS), Nairobi, Kenya

1. The problem statement (last paragraph under the background) needs to come out clearly to state how existing resources and quality systems impact GCLP implementation. Do we want others to learn from us how to implement QMS and better use of resources?
2. First paragraph under methods “This paper describes implementation of Good Clinical Laboratory Practice (GCLP) at the Kenya Medical Research Institute-Center for Microbiology Research (KEMRI-CMR) as part of a quality system to support medical research" fits well with background. I suggest it moves to the background in a summarized form.
3. There is no need to write GCLP in full since it’s already under the background. It’s enough to just abbreviate in all subsequent paragraphs.
4. "The aim was compliance and continuous monitoring to meet international GCLP standards in a way that could be replicated in other research organizations". I suggest the aims go under the background.
5. The author needs to state what the outcome of the baseline assessment was so that this can be compared with the mid-term and exit assessment in terms of nonconformities etc. in the results section. Instead the author talks of what was done following the baseline assessment. This can be covered under the discussion section.
6. No need to mention the names/initials of mentors under Baseline, mid-term and exit assessments section. This may be included as roles for each author at the end of the manuscript.
7. Fig. 1 seems to be communicating information already covered in Fig. 2. I suggest removal of Fig. 1.
8. The first part of conclusion “involving staff in the implementation of QMS” is not very well supported by the results obtained in this study. Mentorship, trainings, active corrective actions following assessments are well supported by the results obtained.
9. "There was also slower progress in reporting of the results, planning of the work" this statement seems to be a repetition in the first paragraph of the discussion.
10. There seems to be minimal improvement in the QSC on ‘conduct of work’ yet this is not mentioned in the discussion.
11. In paragraph 4 of discussion the author seems to be focusing on personalities "His Open door" rather than position e.g. management/leadership.
12. In paragraph four of discussion fortnightly meetings are mentioned but this is not mentioned in methods. Consider having it in methods as part of the strategy that brought success. Also in the results in terms of how many meetings were held in this period compared to previously. This should support the conclusion that involvement of staff was instrumental in QMS implementation.
13. Paragraph five of the discussion need to specify which was mandatory training and which was supplemental training.  

I confirm that I have read this submission and believe that I have an appropriate level of expertise to confirm that it is of an acceptable scientific standard.
